# Mindfulness and Shinrin-Yoku: Potential for Physiological and Psychological Interventions during Uncertain Times

**DOI:** 10.3390/ijerph17249340

**Published:** 2020-12-14

**Authors:** Erica R. Timko Olson, Margaret M. Hansen, Amber Vermeesch

**Affiliations:** 1 School of Nursing, University of Minnesota, Twin Cities Campus, 308 Harvard Street SE, Minneapolis, MN 55455, USA; 2 School of Nursing and Health Professions, University of San Francisco, 2130 Fulton Street, San Francisco, CA 94117, USA; mhansen@usfca.edu; 3 School of Nursing, University of Portland, 5000 N. Willamette Blvd, Portland, OR 97203, USA; vermeesc@up.edu

**Keywords:** mindfulness, Shinrin-yoku, forest bathing, anxiety, mental health, wellbeing

## Abstract

Mindfulness and Shinrin-yoku (SY) translated as *forest bathing*, is potentially effective to alleviate mental health issues related to the COVID-19 pandemic and beyond. The purpose of this article is to provide a translational and pragmatic approach to understanding mindfulness in the context of SY and psychological wellbeing through a rapid review of the literature. The background of mindfulness and SY practice are discussed and the emotional, neuroendocrine, and neurobiological responses are examined. Next, a rapid review of the literature examined six studies, published between 2010 and 2020 to determine what is known regarding the relationship between SY, mindfulness, and psychological wellbeing. The studies included 21–360 participants with a mean age of 20–55 years. The results demonstrated a significant positive correlation between nature, mindfulness, and measures of psychological wellbeing. During uncertain events, including COVID-19, weaving mindfulness with SY may be specifically important to at-risk groups, those experiencing depression, loneliness, and social isolation, and at-risk populations such as college students, veterans, and professionals with high levels of stress. The goal of this review is to provide a thorough background and support of this cost-effective modality to promote overall psychological wellbeing as a preventative measure to those at risk or experiencing psychological illnesses.

## 1. Introduction

The novel coronavirus (COVID-19) dashboard, presented by the Center for Systems Science and Engineering (CSSE) at Johns Hopkins University (JHU), illustrates the brevity of this unprecedented time of global uncertainty. As of 27 October 2020, there are 43,895,968 confirmed cases of COVID-19 in 189 countries with 1,165,455 deaths globally [1]. The confirmed individuals and others collectively have been seriously affected physically, psychologically, spiritually, and/or economically by this pandemic. From a psychological perspective, researchers quickly shined a light on the effects of COVID-19 epidemiological mandates, such as: fear of disease and death, fear of the threat to human existence, heightened state anxiety, social distancing, social isolation, and quarantine [2,3,4,5]. Mental health issues tend to be discounted in various cultures and societies even though mental illness accounts for one-third of adult disabilities worldwide [5]. Mental health interventions are to be actively available at all times and keenly recognized during and after unstable global catastrophes. Mindfulness practice has demonstrated significant positive effects on mental health concerns [6,7,8,9,10,11,12,13] while Shinrin-yoku (SY), translated as *forest bathing* has also demonstrated relaxing and healing effects. When evaluating these two modalities together, would there be a significant effect on emotional wellbeing? The authors’ aims are to provide a background and rapid review of the literature pertaining to mindfulness and SY as interventions to alleviate mental health issues related to the COVID-19 pandemic and beyond.

## 2. Background

### 2.1. Mindfulness

Mindfulness has a long history, dating back to the Buddhist tradition in the first century BC. This history has countless understandings and several historical theories. Mindfulness has varied ways to practice with clinical, philosophical, religious, and secular approaches. With all the complexities within the practice of mindfulness, it is surprisingly not difficult to find common ground in writings and teachings. There are two main purposes of this ancient practice: coping and elimination of suffering, and positive changes in emotional states that lead to changes in behavior [6]. This has been simplified over the centuries to the understanding of “awareness of one′s internal states and surroundings” [7] or “paying attention in a particular way; on purpose, in the present moment, and nonjudgmentally” [8]. When mindfulness is practiced and achieved, there is an improvement in mental, physical, emotional, and spiritual well-being [9].

Health care workers and college students are particularly vulnerable to the negative effects of the COVID-19 pandemic and mindfulness practices have demonstrated positive mitigation of these concerns [10]. Healthcare professionals who practice mindfulness have significant positive effects regarding stress, anxiety, burnout, depression, and psychological distress [11,12,13]. All of these stressors have a negative effect on the provision of patient care, which is of particular importance at this time, with stress, anxiety, and fear so prevalent in all aspects of patients’ lives during this pandemic. College students, in particular, have high rates of stress and fatigue whereas mindfulness encourages engagement, focus, and learning [14].

Mindfulness research has been substantially increasing as an intervention to improve general health and well-being [15]. People are desiring to know and incorporate positive coping strategies [16]. Healthcare providers who participate in mindfulness programs, such as Mindfulness-Based Stress Reduction (MSBR) have experienced an increased quality of life and decreased perceived stress [17]. Research conducted in prison settings supports mindfulness interventions as having positive effects on the physical and mental health of inmates, instructors, and prison staff members [18]. 

Mindfulness provides many opportunities for a variety of practice methods. Several ways to practice include clinical/secular and philosophical/religious approaches. Some examples that have emerged are described here. 

Clinical and secular approaches are used in a variety of settings. Mindfulness-Based Stress Reduction (MBSR) is a formal, 8-week program that is offered at medical centers across the United States. Instructors lead participants through mindfulness meditation techniques with stretches and postures from Hatha yoga. MBSR teaches how to positively handle daily stressors. Mindfulness-Based Cognitive Therapy (MBCT) is MBSR and cognitive therapy as a treatment for chronic depression. MBCT helps people to cope and manage personal thoughts and feelings. There is also Acceptance-Based Behavioral Therapy (ABBT) that includes MBSR and behavioral therapy to treat generalized anxiety disorder (GAD). GAD assists individuals to understand personal thoughts and emotions are normal, expected, and will increase and decrease from moment to moment.

Philosophical and religious approaches have been practiced for thousands of years. The root of mindfulness practice, as we understand it today, is derived directly from Buddhism. In order to practice mindful meditation, it is not required to have an understanding of Buddhism. It is an attempt to pay attention, to listen, and to be aware by simply noticing your breathing. It is gentle, appreciative, and nurturing [19]. An ancient practice in the Christian tradition is that of *lectio divina. Lectio* has roots in the Jewish synagogue, where Rabbis meditated on the Hebrew Scriptures [20]. In the 6th century, St. Benedict discussed the four phases: mind, heart, spirit, and body or read, meditate, pray, and contemplate [21]. We are called to rest in God’s word and allow his vision and value to shape us [20]. Hinduism has deep roots in yoga and Vedic meditation that date back 2500–3500 years. Different types of Hindu mindful meditation today include Mantra Meditation, Transcendental Meditation, and Yoga Meditation. These are all ways to focus and silence the mind using a mantra or focus of attention, concentration, or visualization. 

#### 2.1.1. Mindfulness Emotional Responses

The effect of mindfulness on emotional response has been well documented in the literature ranging from fear conditioning, emotional learning, and reduction of post-traumatic stress disorder symptoms [22]. Roemer, Williston, and Rollins [23] found mindfulness to be associated with enhanced emotional recovery, reduced negative emotional responses, reduction in behavioral avoidance and decreases in emotional regulation difficulties. Roomer and colleagues [23] continue with the interpretation of their findings to indicate the practice of mindfulness is associated with healthy emotion regulation. 

Long and Christian [24], explored the effect of mindfulness as a buffer to ruminative thoughts and negative emotional responses including injustice and retaliation. Their findings are supportive of the buffering effects of mindfulness on ruminative and negative emotional responses which lessen retaliation. This bolster to self-regulation is especially important to health care workers who, through secondary trauma, are likely to experience higher levels of stress leading to negative emotional responses. Long and Christian [24] were among the first researchers to focus on mindful self-regulation in a workplace setting. As the workplace culture shifts to focus on healthier environments, this type of research is imperative to the creation of sustainable and functional workplace environments that support active measures to decrease deleterious circumstances, including retaliation and automaticity. Measures such as this have the potential to increase job satisfaction, retention, and performance through decreasing negative emotional responses. 

The central pillar of Gestalt therapy is awareness which is directly related to the practice of mindfulness [25]. Through increasing mindfulness, the practice of MBSR impacts the modification of emotional responses including those associated with fear response and conditioning [22]. Highlights of this research suggested underlying neural plasticity related to mindfulness practice and its impact on the sensitivity of emotional responses [22]. Practitioners of mindfulness are encouraged to turn toward unpleasant emotions and stimuli rather than away [26]. This is similar to research on increasing the performance and endurance threshold of elite athletes [27]. This research is significant because of the negativity correlated with trait anxiety levels thus suggesting understanding the neural plasticity associated with mindfulness training can be incorporated into successful and sustainably anti-anxiety interventions. Björkstrand et al. [28] found mindfulness training had a positive effect on emotional function through the retention of safety learning protocols for healthy individuals. This has profound implications for the potential treatment of anxiety and trauma-related psychological disorders such as posttraumatic stress disorders [29,30]. 

Relating the effect of mindfulness on fear-based behaviors is highlighted by the research conducted by Orellana-Rios et al. [31] and the importance of developing a mindful presence, cultivating loving-kindness, and inner-self acceptance into daily work activities. Relating the effects of mindfulness on fear-based behaviors is important for the increase in the skills for the self-care strategies toolbox and minimizing the fear of unpleasant circumstances. Incorporating these skills in daily work activities, makes these skills more accessible and sustainable for a larger population. Targeting practitioners and their patients with mindfulness and compassion-oriented practices will be beneficial for all involved. 

#### 2.1.2. Neuroendocrine Responses 

Regarding mindfulness and neuroendocrine effects, the literature [32] suggests mindfulness practice is associated with down-regulation of nuclear factor kappa B, the opposite of chronic stress effects, and thus associated with a reduced risk of inflammation-related disease. This connection is important when considering chronic disease management and prevention, as well as chronic work stressors on employees [33].

Even brief (3-day) mindfulness meditation training has been shown to reduce self-reported perceived stress [34]. However, the increase of salivary cortisol in the same experiment suggests a greater coping effort and cortisol reactivity during evaluative stressors [34]. These results suggest, and are in line with previous research, mindfulness skill acquisition may be mentally demanding but further research is needed to determine the extent of this response. 

#### 2.1.3. Neurobiological Responses

The neurobiological correlates of mindfulness meditation are especially interesting on impulsivity and emotional regulation [35,36,37]. Guendelman et al. [37] explored mindfulness-based regulation interventions to more clearly explore and define a top-down and bottom-up perspective. Mindfulness emotional regulation strategies tend to use a bottom-up approach where the individual regulates emotional response through mindful awareness versus a cognitive reappraisal which relies on a top-bottom mechanism. Guendelman et al. [37] concluded mindfulness practices change the bottom-up emotional regulation system including emotional generation and implicit emotional regulation through long-term meditation training. Although these strategies focus on different aspects including interoceptive-proprioception, sensory-perception, and cognitions and thought process of emotional regulation, the end goal benefits of emotional regulation are clearly accessible to both casual and the expert meditator [37].

### 2.2. Shinrin-Yoku (Forest Bathing)

*Shinrin-yoku* (SY) is a Japanese word first identified by the Director of the Japanese Forestry Agency, Tomohide Akiyama, in 1982 as a method to attract people to visit the Japanese forests. The Japanese characters, or Kanji, for Shinrin-yoku are 森林浴. The first character represents a *forest* (three trees), the second character symbolizes a *wood* and the third character corresponds with *bathe* (flowing water on the left of the character and a valley on the right side of the character). When translated into English, it means *forest bathing* and is similar to the English nuance of *sunbathing*. The practice of slowly and mindfully walking in the forest environment while using all five senses led Japanese scientists to research the physiological and psychological outcomes of experiencing nature [38].

Initially, Miyazaki [38] conducted physiological experiments in 1990 on Japanese participants living in Yakushima because of the plethora of hinoki cedar trees. The effects of SY on the levels of the stress hormone, cortisol, were initially measured because an increase in the number of people living in cities and urban areas were identified as experiencing higher levels of stress. In a decade, new techniques evolved to measure the SY effects on the autonomic nervous system (e.g., brain wave activity) and the incidence of research increased in the 2000s to provide credence to the preventative prescription for enhancing wellbeing and placing less strain on the healthcare system.

Current published research, centered on the physiological health benefits, has increased over the past decade [39,40,41,42]. The physiological health benefits include decreased blood pressure, decreased levels of oxyhemoglobin in the prefrontal cortex, decreased pulse rate, decreased cortisol levels, and decreased heart rate variability (HRV). Most recently, perhaps due to the COVID-19 pandemic, there is a multitude of references pertaining to SY and nature therapy (NT) research by various researchers deeply looking at the relaxing and healing effects of receiving stimuli from natural environments, such as plants, flowers, trees, forests, various woods, and running water in natural environments.

#### 2.2.1. SY Emotional Responses

Focusing on the natural world through an emotional lens of *awe* may have a positive effect on human health and well-being. Flora and fauna also may create safe and secure places for individuals to reflect on emotions, investigate feelings while minimizing the stress response, and provide an opportunity to journal emotions that arise while being exposed to nature and green spaces. According to Bethelmy and Corraliza [43], nature is a commonly acknowledged source of *transcendent emotion* and research has shown nature′s brawn to bring about strong human emotions. The aforementioned researchers generated abstract and operative definitions of a human′s *sublime emotion* toward nature. Based on recent research regarding the feeling of *awe*, Bethelmy and Corraliza [43] developed an instrument to measure *sublime emotion* toward nature and recruited 280 participants from the general population of Madrid, Spain to determine the conceptual definition of *sublime emotion*. Results indicated two conceptual determinants: *awe* and *inspiring energy.* Writers and researchers have identified positive affective effects radiating from the concept of *awe,* as a specific emotional state. Precisely, the *awe* experienced in nature settings reduced human stress-related symptoms and increased overall human wellbeing [44,45].

Exposure to nature, such as green spaces, may have a shielding effect on a human’s mental well-being, such as reported favorable effects on the human stress response. Hunter and colleagues [46] demonstrated 10–30 min of quiet stillness in nature lowers the stress hormone, cortisol. Bratman and associates [47] determined individuals who walked 90 min in nature compared with individuals who walked 90 min along a busy urban road ruminate less about themselves and had a reduction in the activity of the subgenual prefrontal cortex (sgPFC). The sgPFC part of the human brain is an area showing sadness, human withdrawal, and reflection on negative emotional experiences via magnetic resonance imagery (MRI). Furthermore, viewing photographs or videos of nature scenes have a restorative effect, such as fewer negative feelings and increased reported positive moods. Whereas viewing images of urban scenes do not have a soothing effect reported by research participants [48].

#### 2.2.2. SY Neuroendocrine Responses

The neuroendocrine effects of SY are clearly presented in a recent systematic review conducted by Wen and colleagues [49]. Li and co-researchers [50] investigated the effects of SY on cardiovascular health and metabolic factors of 19 middle-aged Japanese male participants. Following a 2.6 km walk (80 min each in the morning and afternoon) in an urban treeless environment and controlling for the exact exposure in a forest environment on two consecutive Saturdays in Japan, the participants’ blood and urine samples were collected before and after each trip. In addition, participants’ blood pressure and pulse rates were measured during the trips, as well as the Japanese version of the Profile of Mood States (POMS) was conducted before, during, and after each walk. The forest bathing environment walks significantly reduced participants’ pulse rate and significantly increased the stamina score and significantly decreased the scores for anxiety, confusion, depression, and fatigue. Showing a tendency toward a decline in urinary adrenaline and a significant drop in urinary dopamine was indicated among the forest bathing program when compared to the urban walking program. Additionally, serum adiponectin, an adipocyte secreted protein that decreases inflammation in the cardiovascular system, was elevated amongst the forest bathing walkers.

Ochiai and research colleagues [51] recruited middle-aged males with high-normal blood pressure in an attempt to study the effects of time spent walking and relaxing in a forest environment on blood pressure and other physiological and psychological factors associated with human stress. The physiological and psychological parameters were measured the same time of day before and circa two hours following the forest walk in order to control for circadian effects. The systolic and diastolic blood pressure readings, as well as urinary adrenaline and serum cortisol, were significantly lower (*p* < 0.05) than baseline following the forest walk. Therefore, these results indicate a forest therapy intervention that has a positive effect on individuals who have been diagnosed with cardiovascular disease.

As mentioned before, SY has been documented as a stress-reducing integrative medicine modality. Kobayashi and colleagues [52] conducted a randomized crossover study and reported the effect of viewing a forest environment versus viewing an urban landscape on male participants (*n* = 348) salivary cortisol concentrations. Each participant sat on a chair for 15 min while viewing a forest environment and then 15 min viewing an urban environment for 15 min. The intervention sites were 34 forest sites and 34 urban sites (city centers) scattered across Japan. The participants’ saliva was collected immediately following each 15-min intervention. Frequency distributions of salivary cortisol intensities, as reported as *mode*, were in a range from 5.73 to 6.55 nmol/L for both groups. However, the participants with a higher salivary cortisol level (>10 nmol/L) were larger in the urban group (*n* = 84) than in the forest environment (*n* = 44). Furthermore, cortisol levels were significantly lower in the forest environment group (mean: 6.88 nmol/L, median: 6.35 nmol/L) than in an urban environment group (mean: 7.98 nmol/L, median: 7.45 nmol/L), and the variation test revealed these differences were statistically significant (*p* < 0.001).

These three studies provide a snapshot of the current research conducted on the SY effects on the neuroendocrine human system. According to the physiological anthropologist and deemed *father of forest bathing,* Miyazaki [53], designed the *back-to-nature-theory* purporting since humans evolved in nature it is where humans feel most comfortable and achieve homeostasis. Miyazaki and associates [54,55,56,57] have extensively conducted and published studies involving physiological markers, such as the autonomic nervous system (heart rate variability), central nervous system (EEG, fMRI and PET scans), endocrine systems (cortisol, adrenaline, nor-adrenaline, immunoglobulin A, and the immune system (natural killer cells).

#### 2.2.3. Neurobiological Responses

Song and research associates [55] examined the effect of viewing real forest landscapes on human prefrontal brain activity. Song et al. [55] set out to conduct this research because there is limited evidence-based research involving brain activity changes when exposed to forest environments. Twenty-nine females (age: 21 ± 1.4 years) were recruited across five forest and five urban environments and were asked to view forest and urban scenes for 15 min each. The oxyhemoglobin (Oxy-hb) levels were constantly measured in the right and left pre-frontal areas of the brain while the participants viewed each scene and the results indicated a lower level of Oxy-hb in the participants’ right pre-frontal lobes while viewing forest landscapes. This area of the human brain is associated with physiological relaxation and these results showed less oxygen uptake in this region of the brain is necessary while viewing nature landscapes. This recently published study supports prior studies [56,57] and studies conducted in a nature therapy laboratory [58,59].

This provided review of the recent research conducted in the areas of neuroendocrine and neurobiological effects associated with SY supports and improves mental health wellbeing. Shinrin-yoku is a living medicine that has been with us for over seven million years as humans evolved in nature (99.9%) versus the time we have spent in urban cities (0.01%) (personal communication, Miyazaki, 2018). Applying Miyazaki′s *Concept of Nature Therapy* [53] to today′s human stressed state during this unprecedented pandemic and global warming times is warranted. The *Concept of Nature Therapy* is a top-down concept that starts with a *stressed state* and from this mindful *stressed state* one looks for exposure to flowers, wood products, and forests with a result in *physiological relaxation* and *immune function recovery*, hence resulting as *preventive medicine.* Weaving mindfulness with this *Concept of Nature Therapy* may be applied to mental health risk groups, such as depression, loneliness, and social isolation during uncertain events as recently experienced at the global level.

## 3. Materials and Methods

A rapid review was conducted to determine what is known regarding the relationship between SY, mindfulness, and measures of psychological wellbeing at this critical time of a global pandemic with COVID-19 (Table 1). A literature search was conducted using the keywords: nature, mindfulness, Shinrin-yoku, psychological wellbeing, and mental health. Inclusion criteria included: peer-reviewed article, published between 2010 and 2020, English language, and mindfulness in nature to measure psychological wellbeing outcomes. Exclusion criteria included: systematic reviews. A total of 29 articles were reviewed with a total of six meeting the inclusion and exclusion criteria.

## 4. Results

### 4.1. Sample and Study Characteristics

The number of subjects in the studies ranged from 21 to 360 with a mean age range between 20.67 and 55 years. The studies not reporting mean age ranges were conducted in a university setting [60], or a work setting [61]. All the research studies measured mindfulness and emotional health outcomes. Two of the studies did not include a connection or relationship with nature questionnaire [61,62] though the studies took place in an outdoor setting. The studies included both survey [60,63,64] and interventional [61,62,65] studies. All studies used convenience sampling and one study did have a small control group [61]. The search also included two pilot studies [61,62].

The purpose of three of the studies was to determine the relationship between nature, mindfulness, and psychological wellbeing outcomes [60,63,64]. The remaining study purposes were to examine the effects of mindfulness training within an outdoor nature environment [61,62,65].

### 4.2. Results

The results showed overall, that there is a significant positive correlation between nature, mindfulness, and measures of psychological wellbeing. Two studies discussed the positive benefits of mindfulness training in nature with the reduction of the burden of illness related to the intervention [61,62] by as much as 40% [61]. Specific psychological conditions such as anxiety, depression, and psychological stress showed significant benefits from mindfulness in nature [60,65]. Connection or relatedness to nature scores was correlated to an increase in mindfulness [60,63,64] and measures psychological wellbeing [60,63,64,65]. Mindfulness was positively correlated with measures of psychological wellbeing [60,61,62,63,65].

### 4.3. Limitations

Research in this area is limited due to small sample sizes, lack of adequate power, at higher than desired attrition rates. The authors of all the studies reported the need for larger sample sizes with randomization to increase power and a greater variety of participants to provide generalizability.

### 4.4. Conclusion

Mindfulness and SY interventions are a cost-effective modality that can be implemented in a variety of settings to promote overall psychological wellbeing to at-risk populations such as college students, veterans, and professionals with high levels of work-related stress.

## 5. Discussion

### Mindfulness and Shinrin-Yoku Connection

*Two are better than one because they have good return for their work: If one falls down, his friend can help him up. But pity the man who falls and has no one to help him up. Also, if two lie down together, they will keep warm. But how can one keep warm alone? Though one may be overpowered, two can defend themselves. A cord of three strands is not quickly broken.* (Ecclesiastes 4:9–12)

Human beings have the innate capacity for health and wellbeing [66]. This capacity is reaching for balance and respite in an ever-changing environment. Yet, nature is also reaching for balance and respite in every changing relationship with humankind. Both humans and nature are seeking, searching, striving to develop the symbiotic relationship that has existed for millennia. Ancient writings reveal spiritual practices of contemplation during challenging times that people would retreat to nature, the deep forest, and the most desolate desert, for days, weeks, months, and even years seeking wellbeing.

We are seeing some increased threats to psychological and emotional wellbeing while nature is showing she may be damaged beyond repair, both in need of healing. Supporting mindfulness in nature will result in a symbiotic relationship that is beneficial to both human wellbeing and nature, rather than destructive of both [67]. Symbiosis is from the Greek word *sumbios*, companion, to *sumioun*, live together, and finally *symbiosis*, a living together [68].

Additionally, mindfulness and SY support environmental well-being. When mindfulness is practiced within SY there is an increase in connection with nature and a reestablishment and strengthening of this bond [69]. With the decline of nature in urban areas, it is even more important to expand the understanding of the benefits to promote a pro-environmental attitude and behavior to strengthen psychological wellbeing.

SY helps to support interconnectedness to nature but it is significantly greater when participants engaged in mindfulness [60,63,64,69]. Mindfulness supports a deeper connection with nature and may support achieving those benefits more efficiently [70]. The health effects of outdoor activities were greatly enhanced when engagement strategies of mindfulness were incorporated into nature activities [62]. Psychological well-being was more likely when people were engaged and interacted with the environment through mindfulness [60,63,64,65,71]. In addition, time in nature can enhance mindfulness while fostering physical, psychological, and spiritual well-being while supporting the development of insight into the reality of the present situation [67].

## 6. Conclusions

In this year of 2020, we have seen over 40 million cases and greater than 1.1 million deaths from COVID-19 [1]. This has caused significant physical, psychological, spiritual, and economic distress to people and the environment, all around the globe. Accessible mental health interventions need to be available and utilized to alleviate further destruction. Mindfulness practice has been shown to improve mental, physical, emotional, and spiritual wellbeing [9]. Techniques can be clinically based, such as MBSR and MBCT [62,65], or philosophically/religiously based from Buddhism, Judaism, Christianity, and Hinduism [19,20,21]. SY experiences have demonstrated a decrease in the stress response [38] and multiple overall health benefits [39,40,41,42]. Mindfulness and SY have shown an increase in positive emotional responses [31,43,44,45,46,47,48], neuroendocrine effects [24,25,26,27,28,29,30,31,32,49,50,51,52,53,54,55,56,57], and even neurobiological responses [35,36,37,55,56,57,58,59]. The greatest effects may be best utilized together with the support of the symbiotic relationship of humanity and nature [60,61,62,63,64,65]. Additional information is available in the supplementary materials. Mindfulness and SY support the health and healing of both self and nature. Psychological wellbeing is greatly enhanced and supported when humanity can slow down, be present, be mindful, rest in the woods, and allow the healing power of nature to perform her healing work from within.

## Figures and Tables

**Table 1:** Review of Literature.

Author/Date	Research Question(s)/Hypotheses	Methodology	Assessment Tools	Analysis & Results	Conclusions	Implications for Future Research
Huynh & Torquati (2019)	The authors proposed a model to describe how stress, connection to nature and mindfulness work together to improve psychological wellbeing.	Survey, 273 in 2016 and 2019, 2016—237 females, 36 males, 2019—87 males. Undergraduate students	Connection to Nature Scale, Mindful Attention Awareness Scale, Philadelphia Mindfulness Sale, Perceived Stress Scale, Satisfaction with Life Scale, Hospital Anxiety and Depression Scale, Positive States of Mind Scale	Descriptive statistics and bivariate correlations were conducted CN was positively correlated with mindful attention, mindful awareness, positive states of mind and life satisfaction. CN was inversely correlated with perceived stress, anxiety and depression. Mindful attention and acceptance were positively associated with positive states of mind and life satisfaction. Mindful awareness was inversely associated with perceived stress and depression and positively corelate with positive states of mind and life satisfaction	Mindfulness significantly mediated the association between connection to nature and psychological well-being and significantly moderated the association between perceived stress and two indicators of psychological well-being.	Replication in an experimental design to determine causation would be warranted
Lücke, Braumandl, Becker, Moeller, Custal, Philipsen & Müller (2019)	Evaluate the efficacy of nature-based mindfulness training in professional with high levels of work-related stress.	Intervention, Pilot, 56 convenience sample, 8 in control group, assessment at baseline, 2 months and 4 months into training	Self-Efficacy (SWE), Sense of Coherence Scale (SOC-L9), Freiburg Mindfulness Inventory (FMI), Symptom Checklist 90 (SCL-90)	Pre-post comparisons, paired t-tests, compared between treatment and control using unpaired t-tests Baseline and 2 months: Significant positive effects in SWE, SOC and FMI and SCL with further subtle improvements through the remaining study time Control group showed a worsening of SOC between T1 and T2.	Participants benefited from the training in level of mindfulness, self-efficacy and sense of coherence. The improvements were small, but the psychiatric burden decreased by 40% where there may be benefits to this intervention that were not measured.	Larger sample size with increased power, experimental design with randomization
Marchand, Klinger, Block, VerMerris, Herrmann, Johnson, Paradiso, Scott & Yabko (2019)	MT/NE might provide a synergistic healing benefit from both the development of mindfulness skills and the benefits of nature exposure.	Intervention, pilot, 21 veterans, 13 male, 8 females, mean age 55, all had at least one psychiatric disorder with 75% having 2 or more. 5 sessions 1x/week over 5 weeks, classroom instruction, mindfulness and sailing,	State train anxiety inventory (STAI), Acceptance and Action Questionnaire II (AAQ-II), Five Facet Mindfulness Questionnaire (FFMQ)	Paired 2-tailed t-tests for pre and post intervention comparison No significant findings in psychological flexibility (AAQ-II) Significant decrease in scores on the STAI and significant increase in scores on the FFMQ 92% enjoyed the experience, 100% stated they felt calm and relaxed, 33% requested more time sailing	Pilot study: determined to be an acceptable and feasible intervention for this population, even those with physical limitations. May positively impact treatment engagement and reduce the burden of illness	Expand the study to both veteran and wider community participants Larger sample with randomization
Sadowdki, Böke, Mettler, Heath & Khoury (2020)	-Assess the relationships between nature relatedness, the five facets of dispositional mindfulness and dimension of subjective wellbeing. -Evaluate the influence of the five facets of dispositional mindfulness as possible mediators between nature relatedness and the dimension of subjective wellbeing	Survey, 250 University students Mean age: 20.67	The Five Facets of Mindfulness Questionnaire, The Nature Relatedness Scale, The Positive and Negative Affect Schedule, The Satisfaction with Life Scale	SPSS, Pearson’s biserial correlations, separate mediation analysis Significant positive associate between nature relatedness and positive affect, life satisfaction, non-reactivity Five facets of dispositional mindfulness were related with overall subjective wellbeing	For female university students, connecting with nature may be more likely to be associated with increased in positive psychological outcomes rather than decreases in negative symptoms. Nature relatedness and elements of wellbeing may be explained by the nonreactivity and observing qualities of mindfulness	Cost effective, accessible modality for implementation in college health centers health services, Further research with larger randomized samples for greater generalizability and power
Wolsko & Lindberg (2013)	Examining the relationship between psychological well-being and the personal experience of connection with nature.	Survey, 256 participants, 2 year community college, mean age 30.11	Connection to Nature (CNS), Mindful Attention Awareness Scale, Flourishing Scale, Scale of Positive and Negative Experience, Subjective Vitality Scale	Correlation and confirmatory factor analysis Higher scores on CNS showed significance with higher levels of trait mindfulness, flourishing, vitality, positive emotion and lower levels of negative emotions Higher levels of trait mindfulness showed significant relationships with flourishing, vitality, positive emotion and lower levels of negative emotions	Significant relationship across all measures including CNS and all measure of psychological wellbeing and mindfulness	Further studies across a variety of samples to determine generalizability
Yeong Choe, Jorgensen & Sheffield (2020)	AIM: Investigate whether the impacts of a commonly used wellbeing intervention (MSBR) are enhanced when combined with the natural environment Hypothesis 1: MBSR achieves the best mental health and wellbeing outcomes when conducted in a natural environment	Intervention, 99 students and staff from a volunteer email list at a university. Mean age 36.35 Divided into 3 groups: natural outdoor, built indoor and indoor environment. Each participated in a 6 week MBSR program of 1 h	Five Facet Mindfulness Questionnaire, Nature Relatedness Scale, Positive and Negative Affect Schedule, Rumination-Reflection Questionnaire, Depression Anxiety Stress Scales	Natural Environment showed greatest adherence, SPSS, ANOVA, MANOVA Positive Affect: Significant Effect over time Negative Affect: Significant impact in natural outdoor environment Rumination: significant interactions between time and environment significant decrease persisted in the outdoor environment Depression: significant for time Anxiety: significant for time Stress: Significant for time and environment	All three groups showed significant changes to mental health and wellbeing. Only the natural outdoor environment group had improved nature connectedness, rumination, reflective attitudes, and stress reduction over the intervention including a one-month follow-up	Continue to study the beneficial effects of nature and mindfulness and an intervention to support well-being, resilience and improve mental health.

## Supplementary Materials

The following are available online at https://www.mdpi.com/1660-4601/17/24/9340/s1.
