# Supplementary material for: Mindfulness and Shinrin-Yoku: Potential for Physiological and Psychological Interventions during Uncertain Times
Source: Int J Environ Res Public Health. 2020 Dec 14;17(24):9340. doi: 10.3390/ijerph17249340 (PMC7764859; doi:10.3390/ijerph17249340)
Supplement: Supplementary file 1 [file ijerph-17-09340-s001.pdf]

**Supplementary Materials:** The following are available online at www.mdpi.com/xxx/s1.

It is helpful to have access to practical resources while seeking solace, respite, calm, healing, and stillness through nature in today's world. The aim of this section is to provide resources leading to the healing qualities of SY, green spaces, and nature therapy. During the COVID-19 pandemic, the use of online resources has become essential for communication and accessing resources by all age groups, therefore, these suggested resources are provided online. The United States Environmental Protection Agency provides an official website of the United States government that provides current information on *Greener Living* [1] and new releases about how to live a greener lifestyle. Various botanical gardens are offering online and in-person nature trails. Currently, the Bloedel Reserve in the State of Washington is offering *Strolls for Wellbeing* [2] as an onsite offering and *Strolls at Home* [3] for those who are sheltering-in-place. The onsite participants are provided a *Strolls for Wellbeing Guidebook* which includes notes for self-reflection.

Mark Coleman offers *Awake in the Wild Nature Meditation* [4], a live and online retreat program for those seeking healing and resilience while being immersed in nature. According to Coleman [4], "*In nature, we can feel a living connection with life all around us. Being outdoors we can taste how we are held and nourished exquisitely by the intricate web of life that is supporting us in every moment, every breath*" p. 1.

The Association for Nature Therapy (ANFT) is known for its leadership in forest therapy guide training and currently provides remote forest therapy guide training in block sections [5]. The ANFT provides a comprehensive online library and recommended readings, as well. The International Forest Therapy Association [6] outlines the health benefits of forest bathing. INFTA, as well as ANFTA, are dedicated organizations that strive to foster partnerships with research, education, and public health around the world. INFTA's mission statement centers on forest therapy being available to all for the promotion of the health and wellbeing of all people worldwide.

INFTA also provides forest therapy guide training and was instrumental in developing the International Core Curriculum on Forest Therapy.

Berry [78] eloquently writes about how the anguish of the world may be diminished by resting in the woods with his poem, *The Peace of Wild Things:*

*When despair for the world grows in me and I wake in the night at the least sound in fear of what my life and my children's lives may be, I go and lie down where the wood drake rests in his beauty on the water, and the great heron feeds. I come into the peace of wild things who do not tax their lives with forethought of grief. I come into the presence of still water. And I feel above me the day-blind stars waiting with their light. For a time, I rest in the grace of the world, and am free* p.1.

This poem is an example of how one may locate, read, and write down inspirational poetry, quotes and sayings about nature in a personal journal and discover the healing powers in order to feel solace in an uncertain world.


1. Greener Living. Available online: https://www.epa.gov/environmental-topics/greener-living (accessed on 29 October 2020).
2. Strolls for Wellbeing. Available online: https://bloedelreserve.org/strolls-for-well-being/ (accessed on 29 October 2020).
3. Strolls at Home. Available online: https://bloedelreserve.org/strolls-at-home/ (accessed on 29 October 2020).
4. Awake in the Wild Nature Meditation. A Live and Online Retreat Program. Available online: https://markcoleman.org/nature-meditation/ (accessed on 29 October 2020).
5. 2020 Forest Therapy Guide Trainings. Available online: https://www.natureandforesttherapy.org/guide-training/training (accessed on 29 October 2020).
6. International Nature and Forest Therapy Alliance (INFTA). Available online: https://infta.net/ (accessed on 29 October 2020).

7. Berry, W. The Peace of Wild Things. Available online: https://onbeing.org/poetry/the-peace-of-wild-things/ (accessed on 01 November 2020).
